# P2RX7 deficiency prevents cognitive deficit, brain atrophy and tauopathy progression in PS19 mice via suppression of extracellular vesicle secretion and mitochondrial toxicity

**DOI:** 10.1002/alz.088010

**Published:** 2025-01-03

**Authors:** Victor B Santos, Mohammad Abdullah, Justice Ellison, Zhi Ruan, Tsuneya Ikezu, Seiko Ikezu

**Affiliations:** ^1^ Mayo Clinic Florida, Jacksonville, FL USA; ^2^ Mayo Clinic, Jacksonville, FL USA

## Abstract

**Background:**

We previously identified the novel mechanism of pathological tau transfer via extracellular vesicles (EVs) in Alzheimer’s disease (AD). Targeting EV secretion to mitigate tau transfer is therefore a promising therapeutic approach for AD. P2X purinoreceptor 7 (P2RX7), an ATP‐gated cationic channel, regulates microvesicle shedding or secretion of multivesicular body‐derived exosomes. We aim to investigate the effect of *P2rx7* deficiency on disease progression in PS19 tauopathy mouse model *in vivo* and by proteomic profiling of brain EVs.

**Method:**

PS19:*P2rx7* ^–/–^ mice at 9 months of age were tested for the fear conditioning, and pathology assessment of the brain atrophy and tau using immunofluorescence against aggregated tau (Alz50) or phosphorylated tau (AT8) and ELISA. Mice were intracranially injected with viral vectors expressing mEmerald‐CD9 in microglia and P301L tau in neurons to visualize the secretion of microglial EV and extracellular tau *in vivo*. Brain EV samples were subjected to proteomic profiling by data independent acquisition mass‐spectrometry.

**Result:**

PS19:*P2rx7* ^–/–^ mice showed significant improvement in contextual and cued memory compared to age‐matched PS19 mice, which was accompanied by preserved cortical and hippocampal volume, and significant reduction of hippocampal tau pathology and pS396 tau in sarkosyl‐insoluble fraction of brain tissues. The number of GFP^+^ microglial EVs were significantly reduced in *P2rx7* ^–/–^ compared to WT mice. Gene ontology pathway analysis of EV proteome showed significant enrichment of mitochondrial pathways in PS19 group compared to WT group, which was significantly downregulated in PS19:*P2rx7* ^–/–^ group.

**Conclusion:**

Our study demonstrated that *P2rx7* deficiency ameliorates cognitive dysfunction and tau pathology development in PS19 mice by dampening microglial EV secretion and EV‐mediated mitochondria transfer, further indicating the therapeutic potential of targeting P2RX7, an EV regulatory molecule, to ameliorate AD progression.

